# Editorial: Coordinated regulation of the balance between stem cell self-renewal and differentiation

**DOI:** 10.3389/fcell.2024.1391626

**Published:** 2024-03-06

**Authors:** Marco Tatullo

**Affiliations:** ^1^ Department of Translational Biomedicine and Neuroscience (DIBRAIN), University of Bari Aldo Moro, Bari, Italy; ^2^ School of Dentistry, University of Dundee, Dundee, United Kingdom; ^3^ Medical Institute for Regeneration and Repairing and Organ Replacement (MIRROR), Interdepartmental Center, University of Bari Aldo Moro, Bari, Italy

**Keywords:** stem cells, cell signaling, cell regulation, regenerative medicine, aging

The balance between stem cell self-renewal and differentiation is a complex mechanism regulated by different factors and signals actively impacting on the biology of the surrounding microenvironment. Understanding this balance is crucial for advancing regenerative medicine and treating diseases directly or indirectly caused by stem cell dysfunction (Pan et al., 2014).

The increasing cellular imbalance during ageing is a complex and multifactorial process that impact on the overall regulation of stem cell metabolism, mainly based on glycolysis, working in quiescent stem cells, and oxidative phosphorylation, working in differentiating stem cells (Tatullo, 2024).

In this landscape, the scientific literature has clearly demonstrated the strategic role of stem cell niches in influencing the ability to balance self-renewal and differentiation towards specific lineages, ensuring tissues homeostasis. Nonetheless, stem cells observed outside their niche often behave differently, showing fate flexibility that presupposes the presence of super-enhancers somewhat able to induce the lineage commitment and plasticity of adult stem cells (Adam et al., 2015).

This Research Topic has investigated some interesting topics on the regulatory mechanisms governing stem cells mitotic division and differentiation in invertebrates and vertebrates, posing intriguing insights on some clinical effects, variously related to molecular, biochemical, genetic or epigenetic cofactors, closely linked to stem cell activity.

During ageing, stem cells develop metabolic changes that can dramatically modify several important pathways, such as cell autophagy and mitophagy. Nonetheless, adult stem cells (ASC) show limited differentiation capabilities, typically restricted to such cell types found in the tissue of origin.

A clinical use-case that well describes the effect of ageing on human tissues is the Hair follicle (HF) homeostasis and the conditions impacting during hair aging, such as oxidative stress, hormonal disorders, inflammation, and DNA damage and repair defects. Liang et al. reported how these factors pose threats to HF cells, especially hair follicle stem cells, and mesenchymal stem cells involved in hair regeneration and pigmentation (Liang et al.).

It is, therefore, evident that declines in stem cell function during ageing are based on a number of alterations in developmental pathways and genetic regulators.

The function of the main genetic regulators of embryogenetic development, the *Hox* genes, a subset of homeobox genes encoding transcription factors that are generally repressed in undifferentiated pluripotent stem cells, has not been well understood in stem cell aging. Steens and Klein have described the HOX genes, and how stem cell-related signaling pathways establish and regulate ASC-specific HOX expression pattern with different temporal-spatial topography (Steens and Klein).

The role of HOX genes is particularly important, as they are involved in the response to epigenetic stresses, which seems to induce stem cell aging in several tissues and cells, such as muscle stem cells (Muscle-SCs). In fact, an altered epigenetic stress response in Muscle-SCs has been linked to limitation of their function and self-renewal, resulting in a limitation in muscle repair and regeneration (Schworer et al., 2016).

Muscle repair and regeneration is an interesting topic, also considering the recent interest towards the production of muscle fibers for cultured meat on a large scale. Katayama et al. have addressed the importance to expand myoblasts in a serum-free medium to avoid cost, ethical, and environmental issues. By using the C2C12 cells as cell model, the authors have demonstrated that such cells differentiate quickly into myotubes and lose their ability to proliferate when plated in a serum-reduced medium; moreover, the addition of methyl-β-cyclodextrin (MβCD) seems to block further differentiation of myoblasts into myotubes, ensuring the balancing towards the proliferative behaviour of myoblasts in a serum-free condition for cultured meat production (Katayama et al.).

These studies have taken into consideration different cell models and molecular patterns that have basically confirmed their applicability *in vitro*; nonetheless, it is strategic to investigate also the regulatory mechanisms underlying the stem cell behaviour during replication or differentiation. There are, in fact, a number of co-factors able to influence the stem cell fate, such as the transduction of mechanical stimuli (Marrellii et al., 2015), the presence of inflammatory conditions (Gardin et al., 2020), or the activity of local enhancers, typically working at nuclear level. In this landscape, functional analyses using intestinal-derived organoids have deepened the knowledge on Angiogenin-4 (Ang4), a member of the ribonuclease A superfamily. Abo et al. have demonstrated that Ang4 influences the intestinal stem cells (ISCs) in a concentration-dependent manner, inducing apoptosis of intestinal epithelial cells (IECs) at high concentration, and promoting the growth of Lgr5^+^ ISCs-based organoids at low concentration (Abo et al.).

In conclusion, the coordinated and multifactorial regulation of the stem cell behaviour is the primary mechanism to control the proper morphogenesis of organs and tissues. In this Research Topic, we have assessed how different regulators participate, by direct or mediate activities, in development of multiple organs, influencing the stem cell behaviour both under *in vitro* experimental conditions and in *in vivo* biomimetic models.
